# Role of Dendritic Cells in Differential Susceptibility to Viral Demyelinating Disease

**DOI:** 10.1371/journal.ppat.0030124

**Published:** 2007-08-24

**Authors:** Wanqiu Hou, Eui Young So, Byung S Kim

**Affiliations:** Department of Microbiology-Immunology, Feinberg School of Medicine, Northwestern University, Chicago, Illinois, United States of America; University of Pennsylvania School of Medicine, United States of America

## Abstract

Although persistent viral diseases are a global health concern, the mechanisms of differential susceptibility to such infections among individuals are unknown. Here, we report that differential interactions between dendritic cells (DCs) and virus are critical in determining resistance versus susceptibility in the Theiler murine encephalomyelitis virus–induced demyelinating disease model of multiple sclerosis. This virus induces a chronic demyelinating disease in susceptible mice, whereas the virus is completely cleared in resistant strains of mice. DCs from susceptible mice are more permissive to viral infection, resulting in severe deficiencies in development, expansion, and function, in contrast to DCs from resistant mice. Although protective prior to viral infection, higher levels of type I interferons (IFNs) and IFN-γ produced by virus-infected DCs from susceptible mice further contribute to the differential inhibition of DC development and function. An increased DC number and/or acquired resistance of DCs to viral infection render susceptible mice resistant to viral persistence and disease progression. Thus, the differential permissiveness of DCs to infectious agents and its subsequent functional and developmental deficiencies determine the outcome of infection- associated diseases. Therefore, arming DCs against viral infection–induced functional decline may provide a useful intervention for chronic infection-associated diseases.

## Author Summary

Many chronic viral diseases are associated with prolonged viral persistence levels, which vary from one individual to another. However, the mechanisms of differential susceptibility to persistent viral infections are unknown. Theiler murine encephalomyelitis virus (TMEV) induces a chronic demyelinating disease similar to multiple sclerosis. In this study, we investigated the potential mechanisms of differential susceptibility to chronic viral persistence in the central nervous system following infection with TMEV. Our results indicate that differential interactions between virus and dendritic cells (DCs), leading to the induction of anti-viral immunity, are critical in determining resistance or susceptibility to virus-induced chronic demyelinating disease. DCs from susceptible mice are much more permissive to viral infection, resulting in severe deficiencies in their development, expansion, and function, whereas DCs from resistant mice are not permissive. Consequently, the DCs in susceptible mice are responsible for poor anti-viral T cell activation, permitting viral persistence and disease development in the host. Interestingly, the administration of additional DCs or pre-activated virus-resistant DCs enables susceptible mice to resist persistent viral infection and disease development. This knowledge may be useful in devising effective means to induce strong anti-viral immune responses, thereby protecting the host from virus-associated chronic diseases caused by persistent viral infection.

## Introduction

Susceptibility to immune-mediated diseases induced by microbial infections varies between individuals. Several genes involved in innate and adaptive immune responses are known to be associated with such differential susceptibility [1,2]. Consequently, different levels and types of immune responses to causative antigens are often correlated with susceptibility/resistance to diseases. However, the underlying immune mechanisms are largely unknown. Therefore, understanding the mechanisms involved in the determination of resistance versus susceptibility is a crucial step towards better disease control. Recent studies have indicated that dendritic cells (DCs) play a critical role in the induction and maintenance of strong immunity to infectious agents [3]. Thus, it is conceivable that the differential responsiveness of DCs to an identical pathogen in resistant and susceptible individuals may lead to disparate levels of protective and/or pathogenic immunity, resulting in differential susceptibility to virus-associated diseases. In this study, we examine this possibility using the Theiler murine encephalomyelitis virus (TMEV)–induced demyelinating disease model for multiple sclerosis (MS).

TMEV is a picornavirus that causes chronic demyelination in the white matter of the central nervous system (CNS) of susceptible mice, such as SJL/J (SJL), following the establishment of persistent infection [4]. This demyelinating disease has immune parameters and histopathology similar to those of chronic progressive MS and thus is extensively studied as an infectious model for MS [4]. However, susceptibility to persistent infection and demyelination varies among inbred strains of mice. For example, in contrast to susceptible SJL mice, resistant strains of mice such as C57BL/6 (B6) are able to completely clear the virus and do not develop late chronic demyelinating disease [5,6]. Many previous studies have indicated that TMEV clearance from the CNS is mainly mediated by CD8^+^ cytotoxic T lymphocytes (CTLs) [7–9], although such T cells may also be pathogenic, particularly during chronic demyelinating disease. The anti-viral CTL response in resistant mice is significantly more rapid and intense compared to that of susceptible mice [10,11], suggesting that this response is critical in preventing the establishment of viral persistence. Similarly, the level of virus capsid–specific CD4^+^ T cell response following viral infection in resistant mice is significantly higher than that in susceptible mice, despite the higher level of overall CD4^+^ T cell infiltration in the CNS of susceptible mice [12]. These results demonstrate that lower levels of initial anti-viral T cell response in susceptible mice may consequently allow the virus to establish chronic persistence. However, the underlying cellular mechanisms involved in such differential levels of anti-viral immune responses between susceptible and resistant mice are not fully understood.

DCs play a central role in the initiation of both innate and adaptive immune responses to infectious agents, including bacteria and viruses [3]. They are distributed in the skin, mucosa, and in lymphoid organs, where they efficiently take up diverse microbial antigens, then process and present them as major histocompatibility complex (MHC)–peptide complexes to T cells. Following the recognition of pathogens, DCs undergo a series of activation processes. Once activated, DCs migrate into the T cell zone of lymphoid organs, where antigen-specific immune cells can be primed. Migration coincides with a maturation process that results in morphological, phenotypic, and functional changes. Moreover, activated DCs have the capacity to recruit and/or activate cells of the innate and adaptive immune systems [3]. However, DC function and its consequent adaptive immune response depend largely upon the interaction between DCs and microbial signals, which can enhance or impair DC function via Toll-like receptors, c-type lectins, CD40, cytokine receptors, or other pathways [3].

Interestingly, several research groups have recently shown the presence of DCs in the meninges and choroid plexus of healthy rodents [13–16]. Moreover, recent studies have demonstrated that local antigen-presenting cells (APCs), possibly DCs, can activate naïve T cells that enter the inflamed CNS [17]. It has also been noted that the DCs associated with CNS vessels are sufficient to prime myelin-reactive T cells in vivo, resulting in CNS inflammation and clinical disease [18]. Thus, it is possible that neurotropic TMEV evades immune recognition and establishes latency by subverting DC function in susceptible strains of mice, but not in resistant mice. In this study, we address the role of DC responses in viral persistence and its consequent demyelinating disease in prototypically susceptible SJL and resistant B6 mice. We sought to answer the following questions: First, is the level of viral infection/replication in DCs from susceptible mice greater than that of resistant mice? Second, are DC differentiation and activation/maturation preferentially impaired in susceptible mice? If this is the case, what are the major mechanisms involved in these differential DC responses following viral infection? Finally, can an increased DC number and/or acquired resistance to viral infection rescue susceptible mice from chronic viral persistence and from developing demyelinating disease?

## Results

### Enhanced TMEV Replication in DCs from Susceptible Mice Blocks DC Maturation

**Figure 1:**
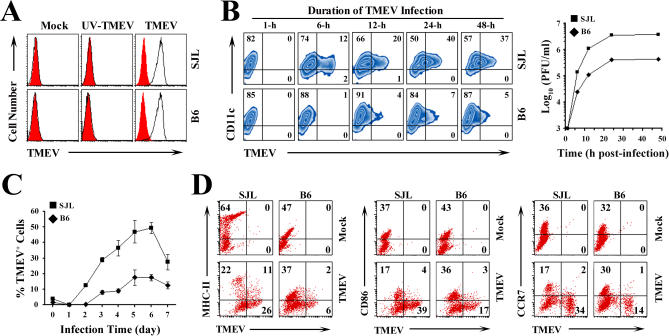
Elevated Viral Replication and Reduced Maturation of SJL DCs following TMEV Infection (A) For the virus binding assay, DCs obtained from a 5-d culture of primary BM cells were incubated with UV-inactivated TMEV or TMEV (MOI 10) for 1 h at 4 °C, fixed with 1% PFA, and stained with anti-TMEV monoclonal antibody (open histogram) or isotype antibody (filled histogram) followed by FITC-conjugated secondary antibody. (B) To determine viral infection and replication levels, DCs were infected at MOI 10 for 1, 6, 12, 24, and 48 h. Expression of TMEV proteins in DCs was detected using anti-TMEV mAb and anti-CD11c antibody. Virus production was assessed by plaque assay. (C) The fraction of virus protein-positive cells was determined at different stages of culture of BMCs. (D) DCs were infected for 24 h and then analyzed for levels of maturation-associated markers (MHC-II, CD86, and CCR7, *y*-axis) and TMEV protein (*x*-axis) by intracellular staining with gated CD11c^+^ cells.

There are significant differences in persistent viral infection and its consequent demyelinating disease between SJL and B6 mice. To examine whether these differences may be associated with differential susceptibility of DCs to TMEV infection, bone marrow (BM) DCs were studied for expression of TMEV antigens and virus production. TMEV was able to bind equally well to DCs from either susceptible SJL or resistant B6 mice (Figure 1A). However, ultraviolet (UV)-inactivated TMEV failed to bind DCs from SJL and B6 mice, which is consistent with the abrogation of receptor-mediated binding of other picornaviruses by UV inactivation [19]. These findings suggest that the binding of TMEV to DCs is not due to non-specific stickiness. In addition, TMEV^+^ cells were detected by 6 h post-infection in the SJL DCs, but not at 1 h post-infection, suggesting that new protein synthesis was required for the detection (Figure 1B). In contrast to SJL DC cells, DC cells from B6 mice were far less susceptible to infection, and the virus yield in single-step growth curves from B6 cultures was approximately one log lower than that from SJL cultures (Figure 1B, right panel). Differences in DC viral infection between SJL and B6 mice were evident throughout several days of culturing BM cells (BMCs) (Figure 1C), despite comparable levels of CD11c in both SJL and B6 DCs (unpublished data). Even on the peak day, day 6, the level of viral infection in SJL DCs was significantly greater (>3-fold) than that of B6 DCs (Figure 1C). It was clear in these experiments that the cells producing viral proteins were CD11c^+^ (Figure 1B), suggesting that DCs produce the majority of infectious virus. In fact, assessment of viral production from isolated CD11c^+^ and CD11c^−^ cells following viral infection confirmed that the great majority of BMCs producing virus proteins (>95%) are CD11c^+^ and the rest (<5%) are CD11c^−^ cells (Figure S1A and S1B). Furthermore, the level of infectious virus produced by CD11c^+^ cells is over 40-fold higher than that produced by CD11c^−^ cells (Figure S1A and S1C). A similar differential virus replication in splenic DCs between these mouse strains was also observed (Figure S1D).

Phenotypic analysis of the infected DC cells suggested that virus preferentially infects immature DCs and prevents their maturation. As shown in Figure 1D, the TMEV^+^ cells in either SJL or B6 cultures were MHC class II^low^, CD86^low^, and CCR7^low^, molecules whose expression is enhanced only during DC maturation. Interestingly, when compared to mock-infected cells, lower levels of expression of MHC class II, CD86, or CCR7 were also observed in infected SJL cultures, even in cells that were not TMEV^+^. For example, 64% of mock-infected cells expressed MHC class II while only 22% of the TMEV^−^ cells in the SJL cultures expressed the molecule. This suggests that the maturation of uninfected DCs is altered in the SJL cultures, possibly indirectly via soluble factors produced by virus-infected cells. This potential bystander effect will be discussed further in the next section. In contrast, infected B6 DC cultures did not show dramatic reductions in their maturation-associated markers. These data strongly suggest that TMEV preferentially replicates in immature DCs and that, in the case of SJL DCs, arrests their maturation.

To exclude the possibility that resistance to persistent TMEV infection in the B6 strain is attributed to the expression of the H-2D^b^ gene [20], DCs from B10 (H-2^b^) and B10.S (H-2^s^) mice, which are relatively resistant to TMEV-induced demyelinating disease compared to susceptible SJL (H-2^s^) mice [21], were also examined. Interestingly, our results show that BM DCs from resistant B10 (H-2^b^) and B10.S (H-2^s^) are similarly resistant to viral infection compared to susceptible SJL (H-2^s^) mice (Figure S2). Thus, these results strongly suggest that differences in viral infection/replication levels in DCs do not reflect the H-2 differences, and that differential susceptibility of DCs correlates closely with susceptibility to TMEV-induced demyelinating disease.

### Cytokines Produced by TMEV-Infected DCs Affect DC Activation

**Figure 2:**
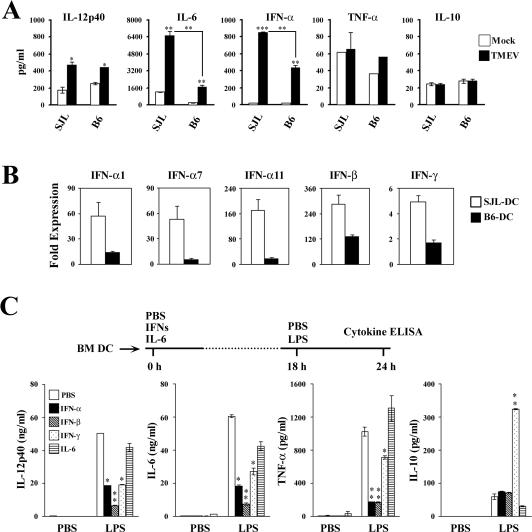
Cytokines Produced by Infected DCs and Their Effects on DC Activation (A) SJL and B6 DCs of a 5-d culture of BMCs were infected with TMEV for 24 h and concentrations of cytokines secreted were determined by ELISA. Statistically significant differences from the values in mock-infected DCs are indicated with asterisks (*, *p* < 0.05; **, *p* < 0.01; ***, *p* < 0.001). (B) SJL and B6 DCs were infected with TMEV for 8 h. Total RNAs from DCs were prepared for quantitative real-time PCR analysis. Data are expressed by fold increases as compared to mock-infected cells. Results are shown as mean plus SD. (C) SJL DCs were treated with IFN-α (1,000 U/ml), IFN-β (1,000 U/ml), IFN-γ (10 ng/ml), IL-6 (100 ng/ml), or left untreated for 24 h. LPS was added for the final 6 h of culture. Cytokine levels in supernatants were determined with ELISA. Statistical differences between the values of untreated and treated DCs are indicated with asterisks (*, *p* < 0.05; **, *p* < 0.01).

To investigate the potential mechanisms for bystander inhibition of DC maturation, cytokines produced in infected DC cultures were examined after 5 d of culture. As shown in Figure 2A, infected cultures produced higher levels of IL-12p40, IL-6, and interferon (IFN)-α than mock-infected controls. Differences in TNF-α and IL-10 were not observed. Interestingly, infected SJL DCs produced significantly more IL-6 and IFN-α than the B6 counterparts. Infection of SJL DCs induced the expression of mRNAs of several IFNs (Figure 2B) with fairly rapid kinetics (8 h post-infection), suggesting that these are direct consequences of the infection. These included type I IFNs (IFN-α1, IFN-α2, IFN-α4, IFN-α5, IFN-α6, IFN-α7, IFN-α9, IFN-α11, IFN-αb, and IFN-β), and a type II IFN (IFN-γ). Figure 2B shows representative examples. In contrast, the levels of other IFNs, such as epsilon, kappa, and lambda, were not significantly different (unpublished data).

The finding that high levels of IFNs are produced by SJL-infected DCs is of importance because these cytokines were found to block the activation of DCs (Figure 2C). When SJL DCs were pretreated with IFN-α , IFN-β, or IFN-γ for 18 h, then stimulated with lipopolysaccharide (LPS) for the final 6 h of culture, DCs produced dramatically less IL-12p40, IL-6, and TNF-α. Interestingly, pretreatment with IFN-γ, but not IFN-α or IFN-β, enhanced IL-10 production, suggesting potential differences among these IFNs in inhibiting DC function (Figure 2C). IL-6, the major cytokine produced by TMEV-infected DCs, had only marginal effects on DC function, which suggests that type I and II IFNs are primarily responsible for the virus-induced inhibitory effects on DC activation.

### IFNs Produced by DCs upon LPS Treatment or TMEV Infection Transiently Limit Viral Infection

**Figure 3:**
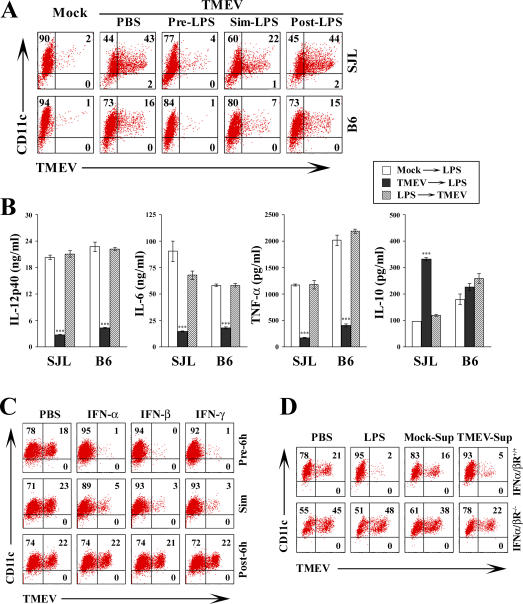
Inhibition of Viral Infection in DCs Pretreated with LPS or IFNs (A) LPS was added 6 h before infection (pre-LPS), simultaneously (sim-LPS), or 18 h after infection (post-LPS). DCs were infected with virus for 24 h and then scored by staining for intracellular viral proteins. (B) DCs were cultured with or without LPS for 6 h, washed twice, and then infected by TMEV for 24 h. After 18 h infection, LPS was added for the final 6 h of culture. Cytokine production was determined with specific ELISA. The differences between the values of mock-infected and TMEV-infected DCs are highly significant (***, *p* < 0.001). (C) IFN-α (100 U/ml), IFN-β (100 U/ml), or IFN-γ (1 ng/ml) was added to SJL DCs 6 h before infection (pre-6 h), simultaneously (sim) with infection, or 6 h after infection (post-6 h). All groups of DCs were infected with TMEV for 24 h. Expression of TMEV proteins in DCs was detected using anti-TMEV mAb and anti-CD11c antibody. (D) Mock- or TMEV-infected SJL DC supernatants were collected after 24 h of infection, and live virus in the supernatants was inactivated with UV. Cell-free supernatants (400 μl/ml) were added to freshly prepared IFN-α/βR-sufficient and -deficient DCs and incubated for 6 h, prior to infection with TMEV for 24 h. Parallel DC cultures were pretreated with LPS or PBS for 6 h. The ability of the fresh DCs to produce viral antigens was detected using anti-TMEV mAb and anti-CD11c antibody.

TMEV was shown to infect immature DCs in Figure 1B, blocking their maturation. In contrast, TMEV does not efficiently infect DCs that have been activated by LPS, a TLR4 ligand (Figure 3A). A variety of TLR ligands, including viral components, are known to induce activation, maturation, and/or cytokine production in DCs [3]. Pretreatment of DCs with LPS greatly reduces the numbers of TMEV^+^ cells in the culture, but treatment of DCs with LPS after infection has no effect. The addition of LPS at the time of infection had only a partial effect. The reduced infection in cells pretreated with LPS was correlated with restored DC cytokine production (Figure 3B). DCs induced by LPS produce the cytokines IL-12p40, IL-6, TNF-α, and IL-10. TMEV preinfection significantly blocked the ability of these cells to produce IL-12p40, IL-6, and TNF-α in response to LPS. In contrast, when DCs were treated first with LPS, cytokine production was not inhibited by the TMEV infection. These results suggest that LPS pretreatment results in inhibition of viral replication and restoration of cytokine production. These patterns were similar in DCs of both SJL and B6 mice, although the levels of viral infection remain higher in SJL DCs compared to B6 DCs. Interestingly, a different pattern was found again with IL-10, and infected SJL DCs produced significantly higher levels of this cytokine in response to LPS.

The findings that virus-infected cells produce IFNs (Figure 2A) and that IFNs reduce the ability of DCs to respond to LPS (Figure 2C) suggest an IFN-mediated inhibition of DC maturation by viral infection. The IFNs produced by infected cells can reduce DC maturation, even in the uninfected cells in the culture, which explains the apparent bystander effect seen in Figure 1D. In addition, the IFNs not only blocked DC maturation, but they also prevented viral infection in pretreated cells (Figure 3C). Furthermore, antibodies to IFNs were actually able to increase the numbers of TMEV^+^ cells in DC cultures (unpublished data). These results are consistent with the possibility that the inhibitory effect of LPS on TMEV infection is mediated by IFNs produced by LPS-treated DCs. It is, therefore, important to note that TMEV-infected SJL DCs produce higher levels of IFNs than infected B6 cells. Even though more virus is produced in the initial infection of SJL DCs, the increased levels of IFNs produced would limit the spread of the infection to neighboring cells. This idea was confirmed by experiments in which supernatants from virus-infected cultures were used to pretreat DC cells (Figure 3D). Pretreatment of fresh DC cells with cell-free culture media from SJL-infected cells dramatically reduced the numbers of TMEV^+^ cells in the new cultures. When DC cells from IFN-α/β receptor knockout (IFN-α/βR) animals were infected with TMEV, the culture supernatants did not effectively block a subsequent infection (Figure 3D, right panel), suggesting that the supernatants inhibit viral infection mainly in a type I IFN–dependent manner. In addition, LPS pretreatment failed to inhibit TMEV replication in IFN-α/βR-deficient DCs (Figure 3D), indicating the involvement of type I IFNs in the resistance of normal DCs acquired by pretreatment with LPS. However, IFN-α/βR-deficient DCs remained partially resistant to viral infection following pretreatment with infected culture supernatants, strongly suggesting that there is a cytokine(s) blocking in a type I IFN–independent manner.

### TMEV Infection Preferentially Inhibits SJL DC Expansion In Vitro

**Figure 4:**
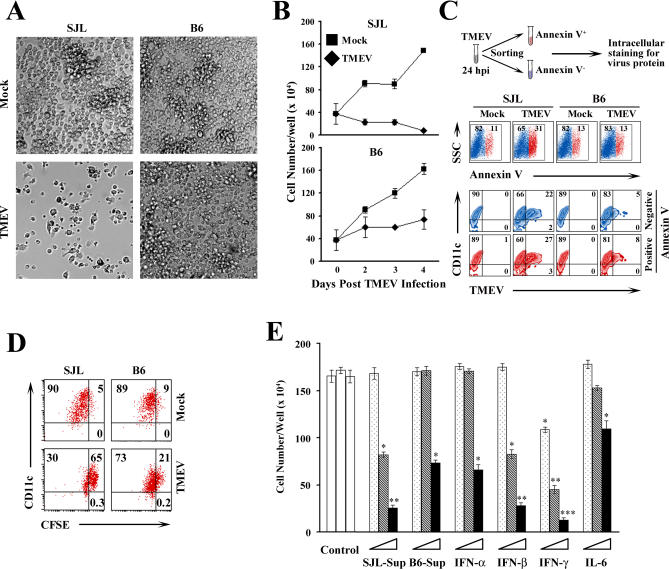
Inhibition of DC Expansion following TMEV Infection via Apoptosis and Growth Arrest (A) BMCs were cultured with GM-CSF for 3 d and then mock-infected or infected with TMEV. Phase contrast micrographs (×100) of BMCs were taken at 3 dpi. (B) BMC cell numbers were determined at day 0 and 2, 3, and 4 dpi. (C) Cells obtained from 3-d BM cultures were infected with TMEV (MOI 10) for 24 h and then labeled with Annexin V. The Annexin V–positive and –negative cells were isolated using an Annexin V MicroBead kit. Cells were then fixed, permeabilized, and stained with anti-TMEV mAb and anti-CD11c antibody. (D) BMCs cultured with GM-CSF for 3 d were stained with CFSE and then infected with TMEV. The decrease of CFSE fluorescence in DCs was determined using anti-CD11c antibody at 3 dpi as shown. (E) BM cells from SJL mice were cultured with 4 μl/ml, 40 μl/ml, or 400 μl/ml of UV-inactivated supernatants from TMEV-infected SJL DCs (SJL-sup) or B6 DCs (B6-sup). BM cells were also cultured with rIFN-α, rIFN-β (10 U/ml, 100 U/ml, and 1,000 U/ml), rIFN-γ (0.1 ng/ml, 1 ng/ml, and 10 ng/ml), or rIL-6 (1 ng/ml, 10 ng/ml, and 100 ng/ml). Increasing concentrations of supernatants or cytokines are denoted by the dotted bars, then striped bars, and,finally, the solid bars. Mock-infected SJL DC and B6 DC supernatant (400 μl/ml) and PBS were included as controls (open bars, respectively). Cell numbers in the cultures were analyzed after incubation in GM-CSF-supplemented medium for 6 d. Statistically significant differences between the numbers of DCs treated with mock-infected supernatant or PBS and DCs treated with TMEV-infected supernatant or cytokines, respectively, are indicated with asterisks (*, *p* < 0.05, **, *p* < 0.01, ***, *p* < 0.001).

Since an adequate number of DCs is necessary to elicit strong innate and adaptive anti-viral immune responses, a substantial reduction of DCs resulting from viral infection may allow viruses to evade immune recognition [22]. To test the possibility that TMEV infection inhibits DC expansion from hematopoietic progenitors, BMCs that were cultured for 3 d in the presence of granulocyte monocyte colony-stimulating factor (GM-CSF) were infected with TMEV, and the rate of DC expansion was subsequently assessed. Only a few small clusters of proliferating cells were observed in virus-infected SJL BMCs at 3 days post-infection (dpi), compared to mock-infected cultures (Figure 4A). In sharp contrast to SJL BMCs, many large aggregates of proliferating cells were evident in virus-infected B6 BMC cultures, similar to that of mock-infected BMCs (Figure 4A). The total number of SJL BMCs infected with TMEV continuously declined during the 4-d culture period, whereas the total number of virus-infected B6 BMCs increased slightly, although the rate of increase was significantly lower than that of mock-infected cultures (Figure 4B). Furthermore, BMCs infected with TMEV failed to differentiate into plasmacytoid DCs or macrophages, as the majority of cells showed CD11c^+^ CD45RA^−^CD11b^+^F4/80^−^ phenotype (unpublished data).

It is conceivable that the suppression of DC expansion is in part due to the apoptosis of BMCs induced by TMEV infection. To evaluate this possibility, we examined the levels of BMC apoptosis at 24, 48, and 72 h post-infection. The proportions of Annexin V–positive cells undergoing apoptosis were significantly higher in TMEV-infected SJL BMCs compared to those in B6 BMCs during the culture periods (shown at 24 h post-infection in Figure 4C; unpublished data). Thus, SJL DCs are substantially more susceptible not only to viral infection but also to virus-induced apoptosis, and this certainly contributes to the failure of DC expansion from hematopoietic progenitors. The preferential apoptosis of SJL DCs may also contribute to the drastic inhibition of SJL DC expansion. However, not all infected DCs are undergoing apoptosis. As shown in the bottom experiment of Figure 4C, when cells infected for 24 h were isolated into apoptotic (Annexin V–positive) and non-apoptotic fractions, a significant number of Annexin V–negative, TMEV^+^ cells were found to be present (22% in SJL versus 5% in B6). It is conceivable that these cells may also be deficient in their expansion. This possibility was confirmed by measuring the loss of carboxyfluorescein diacetate succinimidyl ester stain 3 d after infection (Figure 4D). DCs from SJL mice showed poor proliferation (30% in infected cultures versus 90% in control cultures). In contrast, DCs from B6 mice (73%) showed significant loss of staining intensity, consistent with ongoing cell divisions.

The arrest of DC precursors following infection in SJL cultures is likely to reflect the increased production of IFNs, which is known to block DC expansion [23]. Indeed, when IFN-blocking antibody was included in CFSE experiments, the proliferation of infected SJL DCs was enhanced (unpublished data). It was shown in Figure 3D that culture supernatants from TMEV-infected DCs produced cytokines that blocked further infection, but only when cells from IFNα/βR-sufficient mice were used. Similarly, culture supernatants were able to block the expansion of hematopoietic cell precursors (Figure 4E). The inhibition was dose dependent and the activity of supernatants from infected SJL cells was significantly higher than that of supernatants from infected B6 cells. Type I and II IFNs also similarly reduced cellular expansions. Experiments with IFN-α/βR-deficient DCs indicated that type I IFNs and other inhibitory cytokines, most likely IFN-γ, were responsible for the arresting activity of SJL supernatants (unpublished data). Taken together, these results indicate that BMCs from susceptible SJL mice, but not from resistant B6 mice, are highly permissive to viral infection, leading to virus-induced apoptosis and the production of high levels of regulatory cytokines, including type I IFNs, IFN-γ, and perhaps IL-6, resulting in growth arrest of BMCs.

### Lower DC Numbers Are Present in TMEV-Infected Susceptible SJL Mice

To examine the effects of TMEV infection on SJL and B6 DCs in vivo, phenotypic and functional alterations in DCs from BM, spleen, and CNS of infected mice were analyzed. Interestingly, levels of CD11c^+^ DC populations in the BM and spleen from uninfected naïve SJL mice were significantly lower than those in naïve B6 mice (*p* < 0.01) (Figure S3A). After TMEV infection, the number of B6 DCs in BM rapidly increased at 2 dpi (*p* < 0.05) and then decreased to a basal level at 4 dpi, whereas the number of SJL DCs remained low throughout. Similarly, the number of DCs in the spleens of B6 mice gradually increased (*p* < 0.05), whereas no significant increase was seen in SJL mice. In addition, the levels of DCs undergoing apoptosis in the BM and spleens of virus-infected SJL mice were greater than those of infected B6 mice (Figure S3B). Therefore, lower DC numbers in virus-infected SJL mice are consistent with their differential susceptibility to apoptosis and growth arrest induced by TMEV infection in vitro (Figure 4).

**Figure 5:**
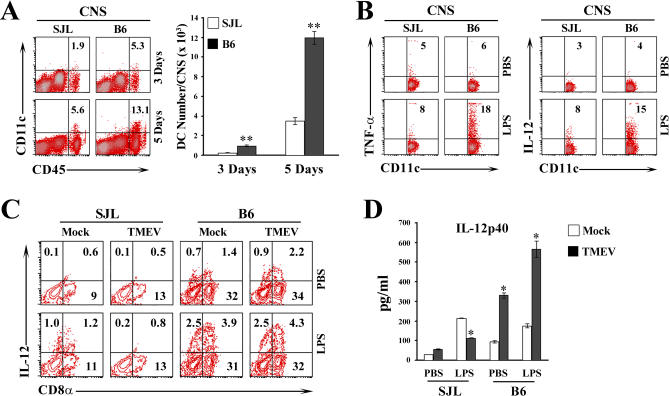
Reduced Number and Ability of DCs to Produce Cytokines in TMEV-Infected SJL Mice (A) The percentage of CD45^hi^CD11c^+^ cells (relative to total CD45^hi^ cells) and the total number of DC cells in the CNS of virus-infected mice (three mice per group) were determined at 3 and 5 dpi. Differences between virus-infected SJL and B6 mice are significant (**, *p* < 0.01). (B) CNS-infiltrating cells from virus-infected mice (three mice per group) at 5 dpi were stimulated with PBS or LPS for 6 h and then stained for CD11c and IL-12. Data are gated on CD11c^+^ cells. The percentage of TNF-α- or IL-12-positive cells relative to total CD11c^+^ cells is indicated. (C) Splenocytes from mock-infected or TMEV-infected mice were prepared at 7 dpi, then stimulated with PBS or LPS for 6 h and stained for CD11c, CD8α and IL-12. Data are shown with gated CD11c^+^ cells. (D) DCs purified from the spleens of mock-infected or TMEV-infected mice at 7 dpi were stimulated with GM-CSF overnight, followed by incubation with PBS or LPS for the final 6 h. IL-12p40 was determined by ELISA. Statistically significant differences from the values of mock-infected mice are indicated with asterisks (*, *p* < 0.05).

The accumulation of DCs in the CNS has been reported in mice with local injections of heat-killed BCG [16] and EAE [24]. Thus, we examined the properties of DCs in the CNS, the virus target organ following TMEV infection. The number of infiltrating CD45^hi^CD11c^+^ DCs in the CNS of infected B6 mice was significantly higher (>2-fold) than that of SJL mice (Figure 5A). In particular, B6 mice showed greater levels of myeloid CD11b^+^ DCs, but decreased levels of lymphoid CD8α^+^ DCs, compared to SJL mice (Figure S4A). In contrast, splenic CD8α^+^ DC levels were lower in SJL mice compared to B6 mice, suggesting a differential distribution of this DC population between resistant and susceptible mice, which may have a role in local immunity to viral infection (Figure S4B). Since TNF-α and/or IL-12 are important cytokines in the migration, accumulation, and activation of DCs involved in the elicitation of anti-viral immunity [3], these cytokines were examined at 5 dpi (Figure 5B). Very low levels of these cytokines were observed in unstimulated DCs from the CNS of TMEV-infected mice. After stimulation with LPS, cytokine production by DCs was enhanced in infected B6, but poorly in SJL, mice. Similarly, DCs from the spleens of virus-infected SJL mice, but not B6 mice, were refractory to cytokine production in response to LPS stimulation (Figure 5C). This result was confirmed with supernatants from isolated splenic DCs from mock- and TMEV-infected mice to exclude the cytokines produced by non-DC population (Figure 5D). Taken together, these results suggest that TMEV infection severely interferes with the survival, CNS accumulation, and function of DCs in susceptible SJL mice, but not in resistant B6 mice.

### TMEV Infection Preferentially Impairs the T Cell Stimulatory Function of SJL DCs

**Figure 6:**
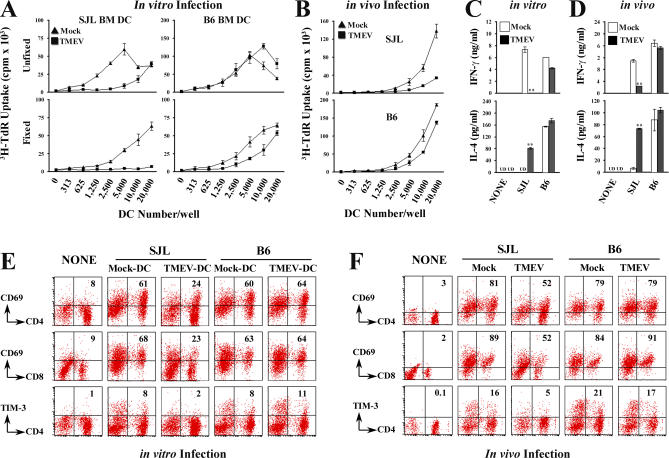
Inhibition of Allogenic T Cell Stimulation by TMEV-Infected DCs BM DCs were infected with TMEV in vitro for 3 d, and then varying numbers of unfixed or fixed BM DCs were cultured with 1 × 10^5^ of naïve T cells from BALB/c mice. To study in vivo infected cells, DCs were isolated from the spleens of mock- or TMEV-infected mice at 7 dpi, then co-cultured with naïve T cells. Cells and culture supernatants were collected after 96 h of co-culture. (A, B) T cell proliferation was measured as the mean ± SD of (^3^H)-thymidine uptake from triplicate cultures. (C, D) IFN-γ and IL-4 produced in culture supernatants (DC:T = 1:10) were determined by ELISA. UD, undetectable. **, significant differences between the values in TMEV- and mock-infected cultures (*p* < 0.01). Data from BM DCs infected in vitro are shown in (C), and those from infected mice in (D). (E, F) The effect of co-culture with infected DCs on the expression of CD69 or TIM-3 on T cells (DC:T = 1:10) was determined by FACS. The numbers represent the percentage of CD69- or TIM-3-positive cells relative to total CD4^+^ or CD8^+^ T cells.

Mixed lymphocyte responses (MLRs) induced by infected DCs were assessed as a measure of DC function. T cells from naïve BALB/c mice were co-cultured with either SJL or B6 BM DCs infected in vitro with virus for 1 d (unpublished data) or 3 d (Figure 6A). The allostimulatory function of virus-infected SJL DCs was severely compromised compared to that of either mock-infected or TMEV-infected B6 DCs. DCs isolated from infected SJL mice were similarly impaired in their ability to drive the MLR (Figure 6B). It is unlikely that this differential reduction in the T cell stimulatory function of SJL DCs is due to infectious virus released from DCs during T cell stimulation, since paraformaldehyde (PFA)-fixed virus-infected SJL DCs similarly failed to stimulate allogeneic T cells (Figure 6A).

Key cytokines produced during allogeneic T cell responses were measured using either BM cells infected in vitro (Figure 6C) or splenic DCs from infected mice (Figure 6D). Interestingly, T cells stimulated with either of these sources of infected SJL DCs failed to produce IFN-γ but gained the ability to produce IL-4, as compared to mock-infected counterparts. In contrast, there was no significant difference in cytokine production when T cells were stimulated with virus-infected or mock-infected B6 DCs. These results strongly suggest that TMEV infection converts SJL DCs from Th1 stimulation to Th2 stimulation, while this infection has a minimal effect on B6 DCs. Since virus-specific Th1 cells are known to be more protective than Th2 cells [7,8], such a switch in CD4^+^ T cell types may also facilitate the preferential persistence of virus in the CNS of susceptible SJL mice. Consistent with the above altered T cell differentiation, the expression levels of CD69, an indicator of T cell activation, and TIM-3, a Th1-associated marker, were reduced when TMEV-infected SJL DCs, but not B6 DCs, were cultured with naïve T cells (Figure 6E). Similar patterns of CD69 and TIM-3 expression on T cells were also observed with splenic DCs from TMEV-infected mice (Figure 6F). These results are consistent with the differential Th1/Th2 stimulation exhibited by SJL and B6 DCs following TMEV infection in vitro as well as in vivo. It is important to note that the expression levels of CD69 on CD8^+^ T cells are similarly reduced after stimulation with infected SJL DCs or DCs from infected mice compared to those from B6 mice (Figure 6E and 6F), suggesting that CD8^+^ T cell stimulation is also compromised.

### DC Administration Confers Protection from Chronic Demyelinating Disease in SJL Mice

**Figure 7:**
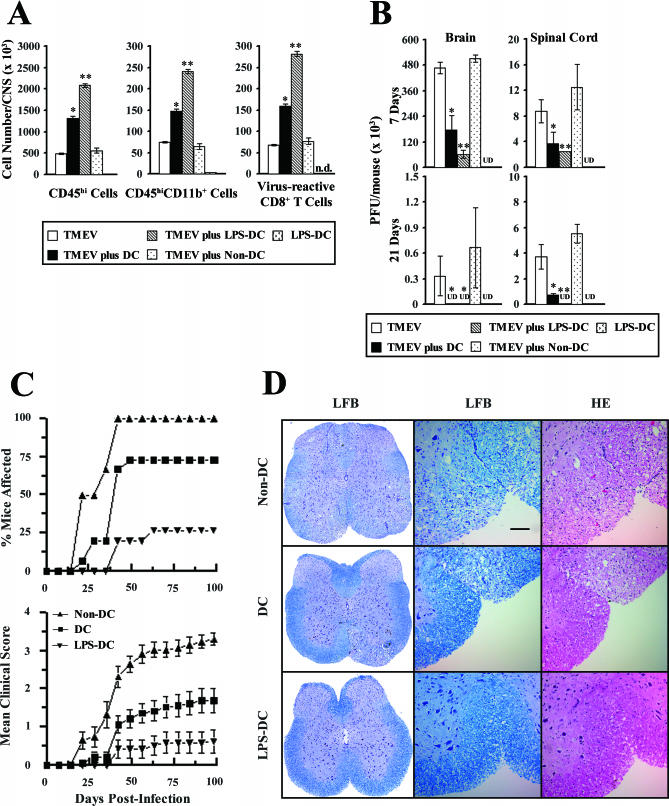
Enhanced CTL Responses, Viral Clearance, and Protection from Demyelinating Disease following DC Transfer into Susceptible Mice BM DCs pretreated with LPS for 6 h (LPS-DCs), untreated DCs, and non-DC splenocytes (1 × 10^5^ cells/per mouse) together with TMEV (1 × 10^6^ pfu/mouse) or virus alone (no DC) were intracerebrally injected into SJL mice. (A) Assessment of levels of CD45^hi^ cells, CD45^hi^CD11b^+^ cells, and virus-reactive CD8^+^ T cells in the CNS of virus-infected mice (three mice per group) at 7 dpi. *, *p* < 0.05 and **, *p* < 0.01 compared to values in mice infected with virus alone. n.d., not done. (B) Level of viral persistence in brains or spinal cords (three mice per group) was assessed by plaque assay at 7 and 21 dpi. UD (undetectable) represents virus levels lower than 24 pfu/per tissue. *, *p* < 0.05 and **, *p* < 0.01 between the experimental groups and the control group infected with virus alone. (C) Disease courses of SJL mice infected with virus plus non-DC splenocytes (*n* = 13), DCs (*n* = 15), or LPS-DCs (*n* = 15) were monitored. Data are presented as the percentage of mice affected with a score of 1 or greater and the mean severity score and standard deviation of their clinical manifestations. (D) Spinal cords of infected mice at 50 dpi were stained with luxol fast blue (LFB) and hematoxylin-eosin (HE), and then examined under a light microscope. The middle and right panels show enlarged images of the left micrographs. Scale bar = 100 μm.

The reduction in DC number and function in SJL mice may contribute to their greater susceptibility to demyelinating infections. Thus, interventions to enhance DC function might reduce the occurrence of disease in these animals. To explore this possibility, SJL mice were intracerebrally infected with TMEV alone or along with syngeneic DCs, LPS-pretreated DCs (LPS-DCs), or non-DC splenocytes (Figure 7). The LPS-DC group was included because LPS-DCs are resistant to TMEV infection and maintain their function (Figure 3A and 3B). At 7 dpi, macrophage levels (CD45^hi^CD11b^+^) and virus-specific IFN-γ-producing CD8^+^ T cells in the CNS were significantly higher in mice infected with virus plus either untreated DCs or LPS-DCs, compared to those in mice infected with virus alone or virus plus non-DC splenocytes (Figure 7A). Control mice receiving LPS-DCs alone failed to accumulate a significant level of leukocytes in the CNS (Figure 7A). The percentage of virus-specific CD8^+^ T cells in the spleens of these mice was low but correlated with the number of CNS-infiltrating T cells. The group that received virus plus LPS-DCs displayed the highest (5.4%) level among all experimental groups (unpublished data). These data indicate that supplying additional DCs, particularly virus-resistant LPS-DCs, facilitates cellular infiltration to the CNS and elicitation of a strong CTL response against the virus.

To relate levels of virus-specific CD8^+^ T cells to viral persistence, the viral load in brains and spinal cords was analyzed by plaque assay (Figure 7B). Levels of virus in the CNS were inversely correlated to the number of virus-specific CD8^+^ T cell levels; viral persistence was significantly lower in mice that received either LPS-DCs or untreated DCs, which resulted in higher CD8^+^ T cell responses. The same patterns of viral persistence were maintained at 21 dpi, although the levels of virus were greatly reduced. More importantly, both the incidence and severity of demyelinating disease (Figure 7C and 7D) were reduced in animals with high levels of the anti-viral CD8^+^ T cell response and viral clearance. Mice receiving LPS-DCs, and to a lesser extent, mice receiving untreated DCs, were more resistant (*p* < 0.001 and *p* < 0.01, respectively) to virally induced demyelinating disease than control mice receiving non-DC splenocytes. Abundant inflammatory foci and areas of myelin stripping were prominent within the white matter of spinal cords from mice receiving non-DC splenocytes (Figure 7D). However, only limited levels of inflammatory cells and restricted myelin damage were observed in spinal cords from mice receiving untreated DCs, and these were less apparent in spinal cords from mice receiving LPS-DCs (Figure 7D). These data indicate that the presence of additional DCs and more effective virus-resistant DCs (LPS-DCs) enable susceptible SJL mice to generate a strong virus-specific CD8^+^ T cell response and control viral persistence, resulting in resistance to demyelinating disease. Therefore, the availability of a high level of functionally intact DCs appears to be a critical factor in determining resistance of the host to virus-induced demyelinating disease, and perhaps similarly to other infection-associated chronic diseases.

## Discussion

**Figure 8:**
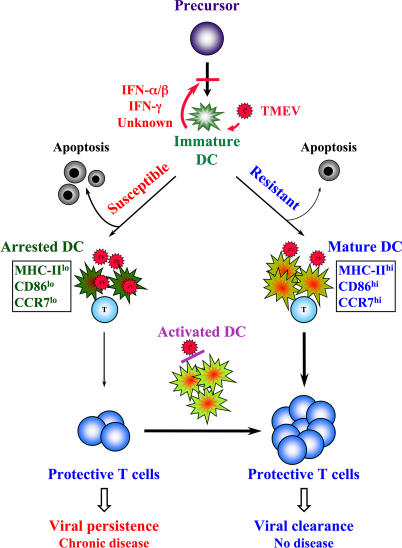
Schematic Presentation of the Potential Role of DCs in Differential Susceptibility to Viral Persistence TMEV preferentially infects immature DCs and induces the production of anti-viral IFN-α/β, IFN-γ, and unknown cytokines. DCs from susceptible mice are more permissive to viral infection and viral-induced apoptosis, and produce higher levels of these cytokines compared to DCs from resistant mice. These cytokines inhibit the development of DCs from BM cells in a dose-dependent manner, leading to arrested maturation and compromised function, while these cytokines can be protective when exposed to DCs prior to viral infection. Consequently, susceptible mice exhibit severely reduced levels of functionally mature DCs for T cell activation, resulting in poor anti-viral T cell responses, and perhaps, preferential non-protective or pathogenic T cell responses. Adoptive transfer of LPS-pre-activated syngeneic DCs, which are resistant to viral infection, renders susceptible mice resistant to viral-induced demyelinating disease by elevating protective T cell responses.

The ability to generate a strong innate and adaptive immune response is critical for the resistance of a host to infectious agents [25–27]. However, many viruses can elude immune responses by an array of diverse pathways, including alterations in antigen presentation, cell apoptosis, and cytokine-mediated signaling [28]. Initial TMEV infection is successfully cleared in resistant mice, but not in susceptible mice, leading to a chronic, MS-like demyelinating disease. Susceptibility to persistent infection and its consequent demyelination is likely dependent upon the balance between viral persistence and host defense immunity. Since DCs are potent APCs, which are essential for strong anti-viral immunity, it would be advantageous for viruses to evade immunological recognition and establish latency by subverting this particular cell population. However, very few systematic comparative studies have been reported regarding the differential susceptibility of DCs to an identical pathogen between resistant and susceptible individuals. Here, for the first time to our knowledge, we provide evidence that the differential susceptibility of DCs to viral infection between resistant and susceptible mice leads to varying levels of DC function in anti-viral immunity and subsequent resistance/susceptibility to chronic demyelinating disease in these mice (Figure 8).

It has recently been shown that generation of the anti-TMEV CD8^+^ T cell response in the CNS requires a BM-derived APC population [29]. Similarly, induction of the T cell response is impaired by DCs infected with foot-and-mouth disease virus, a member of the Picornaviridae [30]. In addition, the altered expression of accessory molecules on DCs upon interaction with human rhinovirus leads to down-modulation of adaptive immune responses during viral infection [31]. In this study, we demonstrate that DCs from SJL mice, but not B6 mice, are extremely permissive to TMEV infection/replication (Figures 1, S1, and S2). The differential susceptibility of DCs to TMEV infection between B6 and SJL mice may not be restricted to this cell type; similar differential susceptibilities to viral infection were also observed in macrophages (unpublished data). Since these cell types are the major TMEV reservoir in the CNS [32], the differential susceptibility of these cells may also contribute to viral persistence in these mice. Nevertheless, resistance of DCs from viral infection appears to be particularly important in facilitating strong anti-viral immunity, leading to viral clearance and protection from developing demyelinating disease.

Furthermore, as diagrammed in Figure 8, our results highlight a series of DC alterations that together may lead to the reduced immune response and viral persistence in susceptible SJL mice, whereas such alterations are not apparent in resistant B6 mice. First, TMEV preferentially infects immature DCs (Figure 1), blocking their differentiation and activation (Figure 4). A subset of the infected DCs undergoes apoptosis, but many arrest and fail to expand and mature. These infected DCs may also contribute to disease pathogenesis by altering the profile of cytokines produced and consequently by stimulating T cell subsets that deviate from those stimulated by intact DCs (Figures 2 and 6). In addition, the infected DCs from SJL mice produce cytokines that lead to bystander effects, inhibiting DC expansion and maturation in response to other stimuli such as LPS (Figures 3 and 5).

TMEV-infected SJL DCs produce significantly higher levels of a variety of type I IFNs and type II IFN-γ compared to B6 DCs (Figure 2). The presence of type I or II IFNs provides protection against TMEV replication, but only when cells are exposed to IFNs prior to viral infection, indicating a transient nature of protection by IFNs (Figure 3). The resistance of TMEV to type I IFNs once infection is established is similar to previous observations in simian virus 40, respiratory syncytial virus, dengue virus type 2, and bovine viral diarrhea virus [33–37]. Thus, IFNs may not only limit the extent of TMEV infection but also provide the opportunity for these viruses to establish persistent infection. In addition, these cytokines also inhibit the production of IL-12p40, IL-6, and TNF-α by DCs in response to LPS stimulation (Figure 2). These results are consistent with the previous report that type I IFNs can negatively regulate IL-12 expression [38]. It is interesting to note that IFN-γ can also negatively regulate the production of these inflammatory cytokines as well as DC expansion from BM precursors (Figures 2 and 4). The potent inhibition of DC expansion and function by IFN-γ was unexpected, as this cytokine is essential for strong anti-viral immunity [7,8]. As far as we know, this novel inhibitory mechanism of IFN-γ on DC function and differentiation has not been previously described.

Notably, the signal pathways involved in the negative regulation by type I and II IFNs may be different since IFN-γ, but not IFN-α/β, displays enhanced IL-10 induction in DCs (Figure 2C). It has been previously reported that IFN-γ can play a negative regulatory role on DC migration and Ag-specific T cell priming in vivo [39]. Since IFN-γ is the major cytokine produced by NK/NKT cells during early innate responses and by CD4^+^ and CD8^+^ T cells during adaptive immune responses following viral infection, IFN-γ may play an important regulatory role by switching DCs on and off for further T cell activation. However, many studies have also indicated that type I and II IFNs play an important role in enhancing DC function in adaptive T cell responses [38]. Therefore, it appears paradoxical that type I and II IFNs can confer DCs with viral resistance (Figure 3), yet these cytokines can also inhibit DC differentiation and function (Figures 2 and 4), which are critical for strong adaptive T cell responses. Our results here strongly suggest that the level of stimulation, duration of stimulation, and timing of exposure to these cytokines following viral infection affect the fate of DC expansion and function.

TMEV infection preferentially abrogates not only maturation and cytokine production by DCs infected with the virus (producing viral proteins), but also bystander DCs (not producing viral proteins) from susceptible SJL mice in culture (Figures 1 and 3). This bystander inhibition is most likely mediated by type I and II IFNs (Figures 2 and 4). Similar deficiencies in DC function are likewise observed in both CNS-associated and splenic DCs from virus-infected susceptible SJL mice (Figure 5). In addition, the T cell stimulatory function of either virus-infected BM DCs or splenic DCs from virus-infected mice is severely compromised, particularly in susceptible SJL mice (Figure 6). Two recent studies using lymphocytic choriomeningitis virus and measles virus have demonstrated that these viral infections prohibit the expansion of DCs in the spleens of mice treated with Fms-like tyrosine kinase 3 ligand [23,40]. Therefore, it appears that inhibition of DC expansion or function may be a common tactic used by various viruses to evade anti-viral T cell responses for the establishment of chronic viral persistence.

It is also noteworthy that significantly higher levels of IL-10 are induced by LPS stimulation in virus-infected SJL DCs than in B6 DCs (Figure 3). IL-10 production is not inhibited after TMEV infection, unlike that of other cytokines, such as IL-12 or TNF-α (Figure 3). High levels of IL-10 may favor Th2 development by TMEV-infected SJL DCs, in contrast to the Th1 development promoted by uninfected DCs or virus-infected B6 DCs (Figure 6). Interestingly, levels of IFN-γ-producing TMEV-specific CD4^+^ T cells are significantly lower, and levels of IL-10-producing CD4^+^ T cells are significantly higher in SJL mice compared to those in B6 mice [12]. Thus, higher IL-10 levels in susceptible SJL mice may promote viral persistence and preferentially elicit regulatory T cells, which further suppress anti-viral responses. Consistent with this notion, HIV-1-infected monocyte-derived DCs do not undergo maturation, but can elicit IL-10 production and T cell regulation [41]. Furthermore, IL-10 may also directly induce immunosuppression, facilitating viral persistence in vivo as recently shown in the lymphocytic choriomeningitis virus model [42,43]. Alternatively, it is possible that TMEV-induced type I IFNs in DCs exert a regulatory role in Th1/2 development; one study showed that exposure to IFN-β during DC-mediated stimulation of naïve Th cells inhibits Th1 cell polarization and promotes the generation of an IL-10-secreting T cell subset [44]. Therefore, it appears that the alteration of IL-10 production is an immune evasion mechanism frequently used by many different pathogenic viruses. Susceptibility to this alteration may also be a critical factor for chronic viral persistence leading to various inflammatory diseases.

In summary, we have demonstrated that DCs from susceptible SJL mice are considerably more permissive to initial viral infection and replication compared to DCs from resistant B6 mice. In addition, TMEV infection preferentially inhibits maturation, activation, and T cell–stimulating function of DCs from susceptible SJL mice in a bystander fashion. The impaired function and reduced number of DCs are likely to add to the poor anti-viral T responses observed in virus-infected susceptible mice, contributing to establishment of persistent infection. It is quite striking to note that such deficiencies and/or abnormalities of DC function are not apparent after TMEV infection in resistant B6 mice. Furthermore, the adoptive transfer of DCs, particularly pre-activated DCs resistant to viral infection, results in the effective clearance of viral persistence and protection from the development of demyelinating disease in susceptible SJL mice (Figure 7). Therefore, the differential responses of DCs to infectious agents may determine the outcome of diseases associated with chronic infections. Our results strongly suggest that increasing DC populations in the target organs and arming them against infections may provide a useful intervention for acute or chronic infection-associated diseases.

## Materials and Methods

### Mice.

Female SJL, B6, and BALB/c mice were purchased from Harlan Sprague Dawley (http://www.harlan.com/). IFN-α/β receptor knockout mice (IFN-α/βR) were kindly provided by Herbert (Skip) Virgin (Washington University, St. Louis, Missouri, United States), and control 129S2/SvPas mice (IFN-α/βR) were purchased from Charles River Laboratories (http://www.criver.com/). Mice (6–8 weeks old) were maintained and used according to protocols approved by the Northwestern University Animal Care and Use Committee.

### Reagents.

The reagents used include IFN-α, IFN-β (both Invitrogen, http://www.invitrogen.com/), and IFN-γ, IL-6 (both PeproTech, http://www.peprotech.com/).

### Virus preparation and infection of mice.

The BeAn strain of TMEV was propagated and titered in baby hamster kidney (BHK) cells grown in DMEM supplemented with 7.5% donor calf serum, as previously described [11]. TMEV or virus-infected DC supernatant was inactivated by exposure to a UV light source for 60 min at 4 °C [45]. The complete inactivation of virus after irradiation was confirmed by the absence of viral plaque formation on BHK-21 cells. Mice were infected by intracerebral inoculation of 30 μl (1 × 10^6^ pfu) of the BeAn strain of TMEV, and clinical symptoms of disease were assessed weekly on the following grading scale as previously described [46]: grade 0 = no clinical signs; grade 1 = mild waddling gait; grade 2 = moderate waddling gait and hind limb paresis; grade 3 = severe hind limb paralysis; grade 4 = severe hindlimb paralysis and loss of righting reflex.

### Generation of BM DCs and expansion.

BM cells were harvested from femurs and tibias of SJL or B6 mice and cultured in RPMI-10 with 20 ng/ml murine rGM-CSF (PeproTech) as described [47]. On day 5, predominantly immature DCs were obtained. For DC expansion experiments, BMCs cultured for 3 d were collected and used as indicated.

### Isolation of CNS infiltrated leukocytes.

Mice were perfused with 30 ml HBSS. Brains and spinal cords were forced through steel mesh and digested at 37 °C for 45 min in HBSS containing 250 μg/ml collagenase type 4 (Worthington Biochemical, http://www.worthington-biochem.com/). CNS infiltrated leukocytes were then enriched on a continuous Percoll (Amersham Biosciences, http://www.gelifesciences.com/) gradient as described [11].

### Virus binding and infection assays.

For the binding assay, BM DCs on day 5 of the culture were incubated with TMEV (MOI 10) for 1 h at 4 °C, fixed with 1% PFA, and stained with monoclonal antibody (mAb) (8C) to the TMEV VP2 capsid protein followed by FITC-conjugated goat anti-mouse Ig (Invitrogen) [6]. For TMEV infection, BMCs were incubated with TMEV (MOI 10) for 1 h at 20 °C with intermittent shaking in RPMI with 0.1% bovine serum albumin (infection medium). Cells were washed twice and then resuspended in complete RPMI-10 medium containing 10% FCS, 20 ng/ml GM-CSF, and 5 × 10^5^ cells per well in a 24-well plate. For the infection assay, monensin (0.7 μl/ml, GolgiStop; BD PharMingen, http://www.bdbiosciences.com/) was added for the final 4 h of infection cultures. Cells were fixed, permeabilized (perm/fix solution, BD PharMingen), blocked with 1% normal goat serum (Invitrogen), and stained with 8C mAb followed by FITC-conjugated secondary antibody or PE-conjugated goat anti-mouse Ig (BD PharMingen). Cells were subsequently stained with anti-CD11c-APC (BD PharMingen). Labeled cells were analyzed on a FACSCalibur flow cytometer. For the maturation-associated marker assay, DCs on day 5 of the culture were infected for 24 h. Cells were stained as described in the infection assay and were further labeled with anti-rat RT1B-FITC (which cross-reacts with mouse I-A^k^ and I-A^s^ alloantigens), anti-I-A^b^-FITC, anti-CD86-FITC (all BD PharMingen), and anti-CCR7-PE (eBioscience, http://www.ebioscience.com/).

### Activation of DCs with LPS and assessment of cytokine production.

In order to determine the effect of LPS on TMEV replication in DCs, 100 ng/ml LPS (Escherichia coli 0111:B4) (Sigma-Aldrich, http://www.sigmaaldrich.com/) were added to DCs on day 5 of the cultures for the indicated time periods. To determine the anti-viral effect of LPS or cytokines, DCs were pretreated for 6 h with these reagents, washed twice, and then infected with TMEV for 24 h. IL-12p40, IL-6, TNF-α, and IL-10 in supernatants of DC cultures at 24 h after infection were measured using BD OptEIA ELISA Set, and IFN-α was determined using an ELISA kit (PBL Biomedical Laboratories, http://www.interferonsource.com/).

### Plaque assay.

Culture supernatants of DCs infected with TMEV for varying time periods were collected for a standard plaque assay on BHK-21 monolayers [11]. In order to analyze viral persistence in vivo, brains and spinal cords were removed from virus-infected mice at 7 or 21 dpi after cardiac perfusion with HBSS. Tissue homogenate was used to perform a standard plaque assay.

### Detection of intracellular cytokines and cytokines in supernatants.

SJL DCs were pretreated with IFN-α, IFN-β, IFN-γ, or IL-6 for 18 h and then stimulated with PBS or 100 ng/ml LPS for 6 h. Supernatants were collected to determine cytokine production and cells were stained for intracellular cytokine production. CNS-infiltrated leukocytes at 5 dpi and splenocytes at 7 dpi were cultured for 6 h with 20 ng/ml GM-CSF in the absence or presence of LPS. Monensin was added for the final 4 h of culture. DCs were fixed, permeabilized, and then stained with anti-CD11c-APC and anti-IL-12p40/p70-PE or anti-TNF-α-PE (both BD PharMingen). To analyze cytokine production by splenic DCs, DCs were positively selected by using anti-CD11c MicroBead (Miltenyi Biotec, http://www.miltenyibiotec.com/) from the spleens of virus-infected mice at 7 dpi. Cultures were further incubated for 24 h in medium containing 20 ng/ml GM-CSF, with or without LPS (100 ng/ml) during the last 6 h of culture. Supernatants were collected to determine IL-12p40 levels. For the cytokine production assay from T cell cultures, supernatants were collected 96 h later. IFN-γ and IL-4 were measured using BD OptEIA ELISA Set.

### Real-time PCR.

Total RNA was isolated from SJL DCs or B6 DCs infected with or without TMEV for 8 h, using Trizol (GIBCO BRL, http://www.invitrogen.com/) by the manufacturer's instructions. Reverse transcription was performed using 1–3 μg of total RNA. The sense and antisense primer sequences used are as follows: IFN-α1 (5-TAGGCTCTGTGCTTTCCTGATGGT-3 and 5-GGTTGCATTCCAAGCAGCAGAT GA-3), IFN-α7 (5-AAGGACTCATCTGCTGCTTGGGAT-3 and 5-ACTCAATCTTGCCAGCAACT TGGC-3), IFN-α11 (5-TCAAACACACAGTCCAGAGAGCCA-3 and 5-ATCCAAAGTCCTGC CTGTCCTTCA-3), IFN-β (5-GCTCGAGCCCCAGGGAATGAACAACAGG TGG-3 and 5-GCTCGAGTCCCTGGGGGTTTTGGAAGTTTCT-3), IFN-γ (5-ACTGGCAA AAGGAT GGTGAC-3 and 5-TGAGCTCATTGAATGCTTGG-3), and GADPH (5-AACTTTGGC ATTGTGGAAGG-3 and 5-ACACATTGGGGGTAGGAACA-3). Specific RNA messages were amplified in iCycler SYBR green I mastermix (Bio-Rad, http://www.bio-rad.com/), using an iCycler (Bio-Rad). Quantification of the genes of interest was normalized to GAPDH in each cDNA and was expressed by fold increase compared with mock-infected DCs. All real-time PCR was performed in triplicate.

### Apoptosis assays.

For the BM DC apoptosis assay, DCs obtained from a 3-d BM culture were infected with TMEV for 24 h and then either stained with FITC-conjugated Annexin V (Invitrogen) or labeled with Annexin V MicroBead (Miltenyi Biotec) for cell separation. The Annexin V–positive and –negative cells were separated, fixed, permeabilized, and stained with anti-TMEV mAb and anti-CD11c antibody.

### Proliferation and MLR assays.

BMCs on day 3 of the culture were labeled with CFSE (Molecular Probes, http://probes.invitrogen.com/) and then infected with TMEV. After 3 d of incubation, cells were stained with anti-CD11c-APC and the level of proliferation was assessed using FACS. Primary allogeneic MLR was performed with mock-infected or virus-infected BM DCs at 3 dpi that were unfixed or fixed with 0.1% PFA as described [48]. DCs from the spleens of mock-infected or TMEV-infected mice were positively selected by using anti-CD11c MicroBead (Miltenyi Biotec) at 7 dpi. Responder T cells from naïve BALB/c mice were enriched from spleens by depleting adherent cells at 37 °C for 2 h and then negatively selected by using anti-CD19 MicroBead (Miltenyi Biotec) to remove B cells. Varying numbers of DCs were cocultured with purified T cells (10^5^/well) in 200 μl of RPMI-10. The cultures were then incubated for 96 h, followed by an 18-h pulse with 1 μCi of [^3^H]-thymidine/well (Amersham Biosciences). For the T cell activation assay, cells were collected 96 h later and stained with anti-CD4-APC, anti-CD8-FITC, and anti-CD69-PE (all BD PharMingen) or anti-TIM-3-PE (eBioscience).

### Adoptive DC transfer in combination with TMEV infection.

SJL DCs cultured for 5 d were stimulated with or without 100 ng/ml LPS for 6 h (LPS-DCs) and then washed twice. The cell pellets were then resuspended in DMEM and incubated with TMEV (MOI 10) for 30 min at 4 °C. Non-DC splenocytes depleted of DCs by anti-CD11c MicroBead (Miltenyi Biotec) were used as an irrelevant cell type control. Mice were infected by intracerebral inoculation of 30 μl containing 1 × 10^5^ cells and/or 1 × 10^6^ pfu TMEV. In addition, mice were injected with LPS-DCs alone as an additional control group.

### Histology.

Spinal cords were removed at 50 dpi after perfusion as described above, fixed in 10% buffered PFA overnight, and then embedded in paraffin. Sections were stained with luxol fast blue (myelin stain) and hematoxylin-eosin, and then analyzed by microscopy. Ten different sections of the lumbar region of the spinal cord of individual mice were evaluated for demyelination. A representative set of micrographs is shown.

### Statistical analysis.

Results are expressed as mean ± SD, where applicable. The Student's *t* test was used to compare two independent groups. The differences in mean severity scores of the two experimental groups were compared between 21 and 98 dpi by using a paired Student's *t* test. Multiple group comparisons were done by a one-way analysis of variance (ANOVA) with Tukey post-hoc analysis. *p*-Values < 0.05 were considered to be significant.

## Supporting Information

Figure S1 CD11c^+^ Cells Are the Major Virus-Infected Population in the BM Cell Culture (A) Experimental scheme of cell separation and determination of viral infection levels of subpopulations in BM cultures. Five-day cultured BM cells were infected with TMEV (MOI 10) for 1 h at 20 °C. After washing, cells were separated using anti-CD11c-coated magnetic beads (Miltenyi Biotec). Separated CD11c^+^ and CD11c^−^ cells and unseparated BMCs from aliquots of the same BMC preparation were then cultured for 24 h with 20 ng/ml GM-CSF.
(B) Expression of TMEV proteins in cells was determined using flow cytometry after staining with anti-TMEV mAb and anti-CD11c antibody.
(C) Virus titers in the culture supernatants of the separated cells were analyzed by plaque assay. The differences between the values of separated and unseparated cells are extremely significant (***, *p* < 0.001), as indicated by asterisks.
(D) Purified splenic DCs by positive selection using anti-CD11c-coated MicroBeads were infected with TMEV (MOI 10) for 24 h. The culture supernatants were collected 24 h post-infection and the level of replicating TMEV was determined by plaque assay. Statistical differences compared to the values of SJL DCs are indicated by asterisks (*, *p* < 0.05). UD, undetectable.
(7.1 MB PDF)

Figure S2 MHC Haplotype H-2^b^ Is Not Required for DC Resistance to Viral Infection BM DCs from B10.S and their counterpart control mice (B10 and SJL) were infected with TMEV (MOI 10) for 24 h. Expression of TMEV proteins was assessed using anti-TMEV mAb and anti-CD11c antibody. DCs from B10.S mice exhibiting susceptible H-2^s^ display resistance to TMEV infection similar to that of B10 mice carrying resistant H-2^b^ haplotype.
(10.5 MB PDF)

Figure S3 Lower DC Numbers Are Present in Virus-Infected Susceptible SJL Mice (A) The level of CD11c^+^ cells per femur/tibia or spleen is shown. Each symbol represents the cell frequency or number in each mouse. The average of each group is denoted with horizontal bar.
(B) BM cells and splenocytes from mock-infected (filled histogram) or TMEV-infected (open histogram) mice were stained with CD11c, PI, and Annexin V at 2, 4, and 7 dpi. Live DCs (CD11c^+^ PI^−^ cells) were assessed with Annexin V. Relative mean fluorescence intensity of DCs from TMEV-infected mice, differing from the values of DCs from mock-infected mice, is depicted on graphs.
(4.6 MB PDF)

Figure S4 DC Subpopulation Distribution Is Different between TMEV-Infected SJL and B6 Mice (A) The expression of CD45, CD11c and CD11b, CD8α, or CD45RA on CNS-infiltrating cells was assessed at 5 dpi. Data represent the cells gated with CD45^hi^ cells. The percentage of CD11b-, CD8α-, or CD45RA-positive cells within the CD45^hi^CD11c^+^ population was shown in the FACS plots.
(B) The expression of CD8α and CD45RA molecules on gated CD11c^+^ splenocytes was determined at 7 dpi by FACS.
(3.4 MB PDF)

## Abbreviations

APC: antigen-presenting cell
B6: C57BL/6
BM: bone marrow
BMC: bone marrow cell
CNS: central nervous system
CSFE: carboxyfluorescein diacetate succinimidyl ester
CTL: cytotoxic T lymphocyte
DC: dendritic cell
dpi: days post-infection
GM-CSF: granulocyte monocyte colony-stimulating factor
IFN: interferon
LPS: lipopolysaccharide
mAb: monoclonal antibody
MHC: major histocompatibility complex
MLR: mixed lymphocyte response
MS: multiple sclerosis
PFA: paraformaldehyde.
SJL: SJL/J
TMEV: Theiler murine encephalomyelitis virus
UV: ultraviolet
