# Supplementary material for: Role of Dendritic Cells in Differential Susceptibility to Viral Demyelinating Disease
Source: PLoS Pathog. 2007 Aug 24;3(8):e124. doi: 10.1371/journal.ppat.0030124 (PMC1950949; doi:10.1371/journal.ppat.0030124)
Supplement: Supplementary file 1 [file ppat.0030124.sg001.pdf]

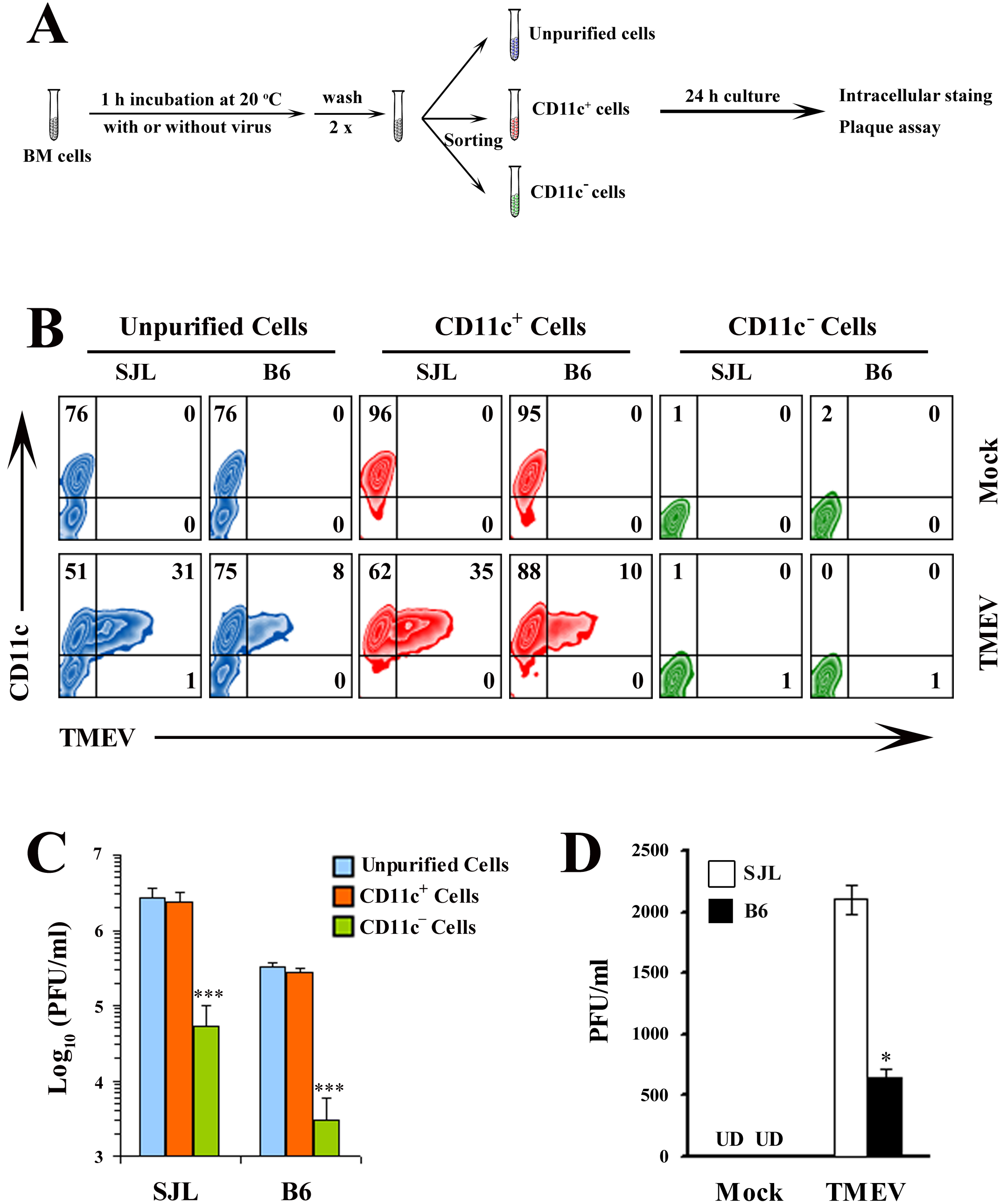

A
BM cells
1 h incubation at 20 °C
with or without virus
wash
2 x
Sorting
Unpurified cells
CD11c+ cells
CD11c- cells
24 h culture
Intracellular staing
Plaque assay
B
Unpurified Cells
CD11c+ Cells
CD11c- Cells
SJL
B6
Mock
TMEV
CD11c
TMEV
76 0 0
76 0 0
96 0 0
95 0 0
1 0 0
2 0 0
51 31 1
75 8 0
62 35 0
88 10 0
1 0 1
0 0 1
C
Log10 (PFU/ml)
Unpurified Cells
CD11c+ Cells
CD11c- Cells
***
***
SJL
B6
D
PFU/ml
SJL
B6
UD UD
*
Mock
TMEV
