# Supplementary material for: Role of Dendritic Cells in Differential Susceptibility to Viral Demyelinating Disease
Source: PLoS Pathog. 2007 Aug 24;3(8):e124. doi: 10.1371/journal.ppat.0030124 (PMC1950949; doi:10.1371/journal.ppat.0030124)
Supplement: Supplementary file 2 [file ppat.0030124.sg002.pdf]

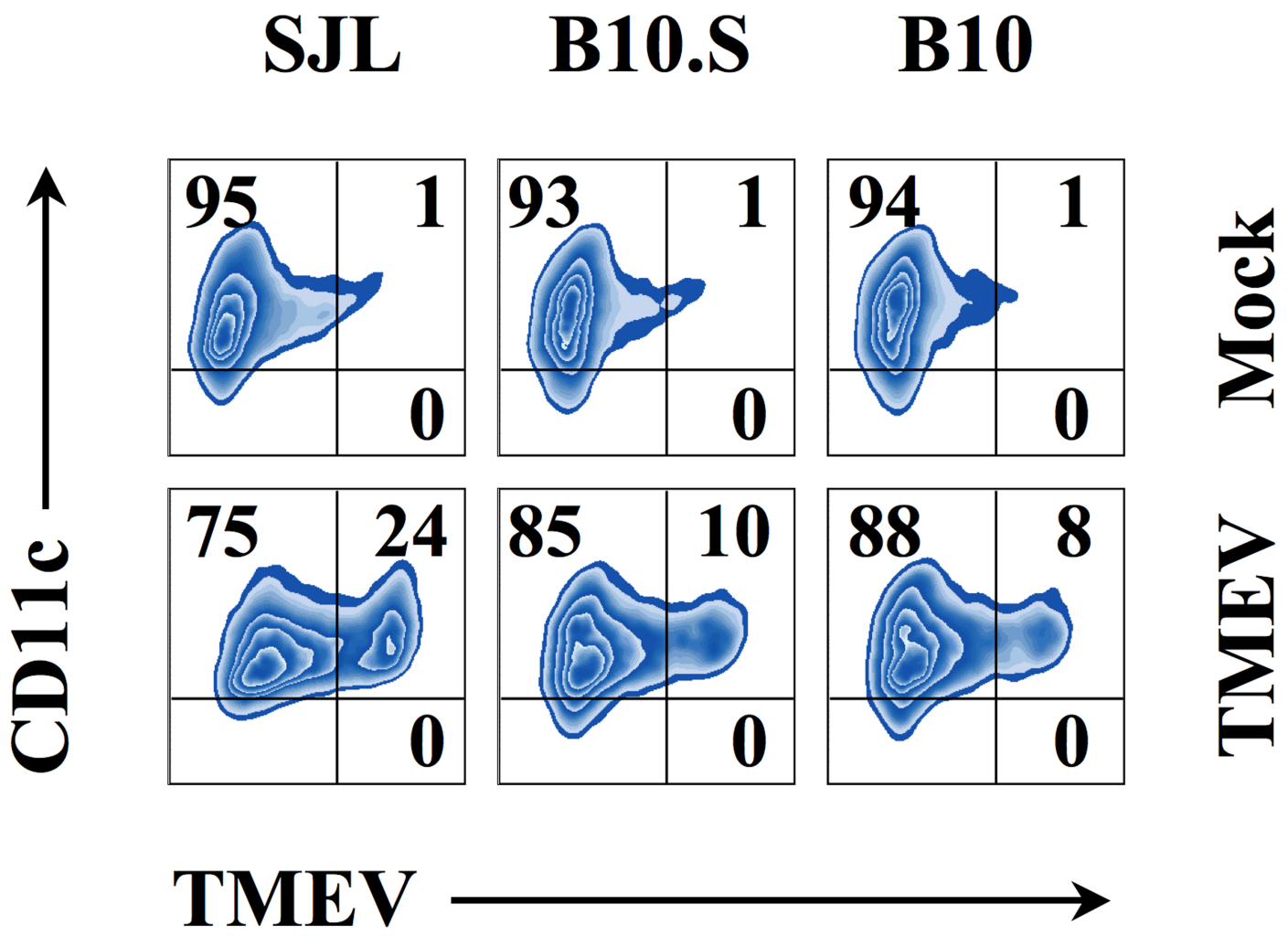
SJL
B10.S
B10
95
1
0
93
1
0
94
1
0
75
24
0
85
10
0
88
8
0
Mock
TMEV
CD11c
TMEV
