# Supplementary material for: Role of Dendritic Cells in Differential Susceptibility to Viral Demyelinating Disease
Source: PLoS Pathog. 2007 Aug 24;3(8):e124. doi: 10.1371/journal.ppat.0030124 (PMC1950949; doi:10.1371/journal.ppat.0030124)
Supplement: Supplementary file 3 [file ppat.0030124.sg003.pdf]

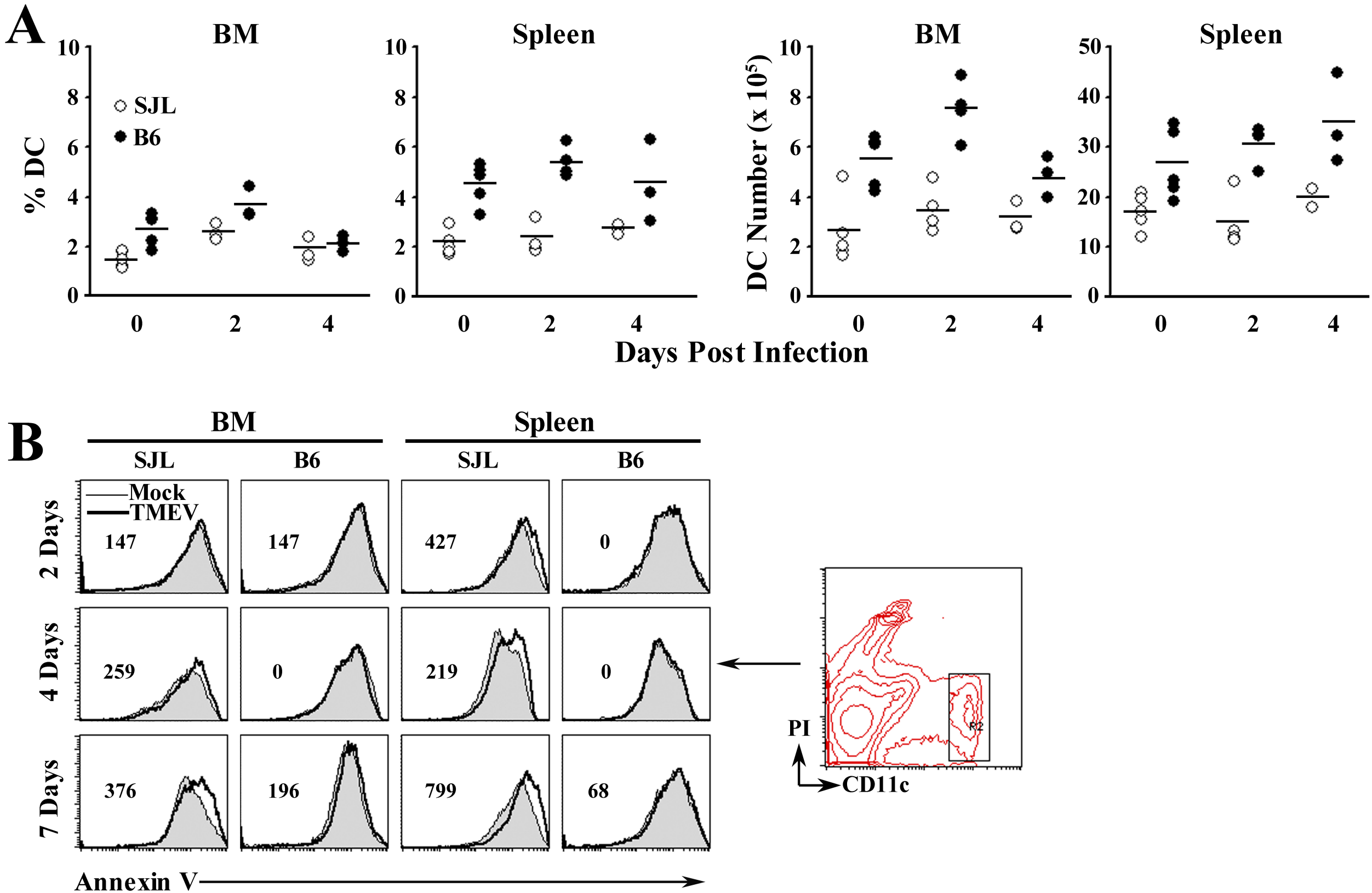
A
BM
Spleen
BM
Spleen
% DC
SJL
B6
DC Number (x 10⁵)
Days Post Infection
B
BM
Spleen
SJL
B6
SJL
B6
Mock
TMEV
2 Days
4 Days
7 Days
147
147
427
0
259
0
219
0
376
196
799
68
Annexin V
PI
CD11c
R2
