# Supplementary material for: Role of Dendritic Cells in Differential Susceptibility to Viral Demyelinating Disease
Source: PLoS Pathog. 2007 Aug 24;3(8):e124. doi: 10.1371/journal.ppat.0030124 (PMC1950949; doi:10.1371/journal.ppat.0030124)
Supplement: Supplementary file 4 [file ppat.0030124.sg004.pdf]

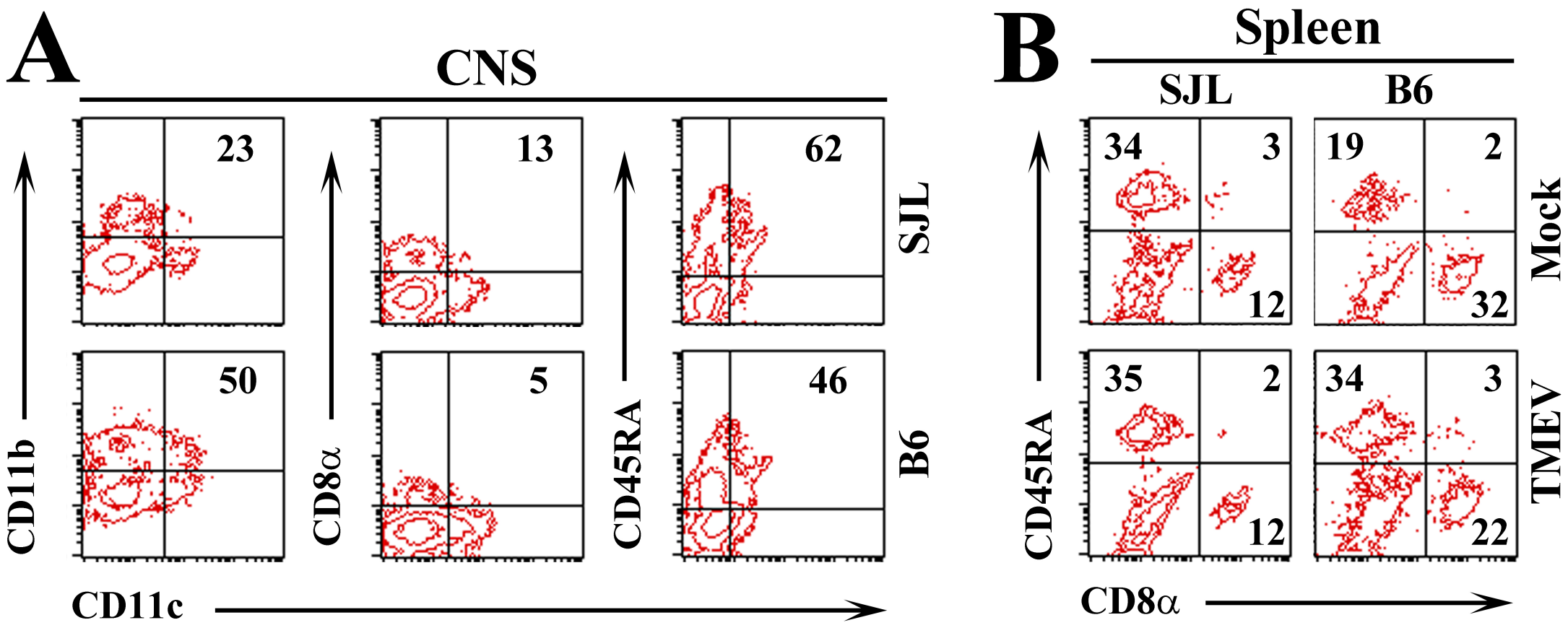

A
CNS
CD11b
CD11c
CD8α
CD45RA
23
13
62
50
5
46
SJL
B6
B
Spleen
SJL
B6
CD45RA
CD8α
34
3
12
19
2
32
35
2
12
34
3
22
Mock
TMEV
